# Integrated approach in the control and management of skin neglected tropical diseases in three health districts of Côte d’Ivoire

**DOI:** 10.1186/s12889-020-08632-6

**Published:** 2020-04-17

**Authors:** Aboa Paul Koffi, Théodore Ange Kouakou Yao, Yves Thierry Barogui, Gabriel Diez, Simplice Djakeaux, Marie Hélène Zahiri, Ghislain Emmanuel Sopoh, Silvia Santos, Kingsley Bampoe Asiedu, Roch Christian Johnson, Henri Assé

**Affiliations:** 1 Programme National de Lutte contre l’Ulcère de Buruli, Abidjan, Côte d’Ivoire; 2 Centre Inter Facultaire de Formation et de Recherche en Environnement pour le Développement Durable, Université d’Abomey Calavi, BP: 2733, Abomey-Calavi, Bénin; 3 Fondation Anesvad, Bilbao, Spain; 4 Programme National d’Elimination de la Lèpre, Abidjan, Côte d’Ivoire; 5 Institut Régional de Santé Publique, Université d’Abomey Calavi, Ouidah, Bénin; 6 World Health Organization, Geneva, Switzerland; 7 Fondation Raoul Follereau, Fondation Raoul Follereau, 31 rue de Dantzig, 75015, Paris, France

**Keywords:** Neglected tropical diseases - integration, Skin diseases - Côte d’Ivoire

## Abstract

**Background:**

Neglected tropical diseases (NTDs) comprise 20 communicable diseases that are prevalent in rural poor and remote communities with less access to the health system. For effective and efficient control, the WHO recommends that affected countries implement integrated control interventions that take into account the different co-endemic NTDs in the same community. However, implementing these integrated interventions involving several diseases with different etiologies, requiring different control approaches and driven by different vertical programs, remains a challenge. We report here the results and lessons learned from a pilot test of this integrated approach based on integrated screening of skin diseases in three co-endemic health districts of Côte d’Ivoire, a West African country endemic for Buruli ulcer, leprosy and yaw.

**Method:**

This cross-sectional study took place from April 2016 to March 2017 in 3 districts of Côte d’Ivoire co-endemic for BU, leprosy and yaws. The study was carried out in 6 stages: identification of potentially co-endemic communities; stakeholder training; social mobilization; mobile medical consultations; case detection and management; and a review meeting.

**Results:**

We included in the study all patients with skin signs and symptoms at the screening stage who voluntarily accepted screening.

In total, 2310 persons screened had skin lesions at the screening stage. Among them, 07 cases were diagnosed with Buruli ulcer. There were 30 leprosy cases and 15 yaws detected. Other types of ulcerations and skin conditions have been identified and represent the majority of cases detected. We learned from this pilot experience that integration can be successfully implemented in co-endemic communities in Côte d’Ivoire. Health workers are motivated and available to implement integrated interventions instead of interventions focused on a single disease. However, it is essential to provide capacity building, a minimum of drugs and consumables for the care of the patients identified, as well as follow-up of identified patients, including those with other skin conditions.

**Conclusions:**

The results of this study show that the integration of activities can be successfully implemented in co-endemic communities under the condition of staff capacity building and minimal care of identified patients.

## Background

As defined by the WHO, neglected tropical diseases (NTDs) currently include 20 prevalent communicable diseases in tropical and subtropical areas. These diseases affect more than a billion people in the world [1, 2]. The WHO classified NTDs into two groups: those that are amenable to preventive chemotherapy (PC-NTDs) and those that are addressed through case management (CM-NTDs) [3]. In addition, there are NTDs (such as BU, leprosy, yaws, cutaneous leishmaniasis and mycetoma, onchocerciasis, lymphatic filariasis) that have in common easily recognizable skin manifestations [3]. The WHO target by year 2020 for the three skin NTDs (leprosy, BU and yaws) is elimination as a public health problem, control and eradication in all endemic countries [3]. Thus, examining the skin of people in communities or schools offers the opportunity to identify patients and detect several diseases in one visit.

Côte d’Ivoire is a West African country endemic for BU, leprosy and yaws [4–8]. While the situation of leprosy and BU is relatively well documented, data on yaws are limited [7, 9].

According to official statistics of the Ministry of Health and Public Hygiene of Côte d’Ivoire, the epidemiological trend of new cases of leprosy and BU has been declining. A total of 1169 leprosy cases were reported in 2013, compared to 567 in 2019 [10], and 1039 cases of BU were detected in 2013, compared to 251 in 2019 [11]. This decrease in the number of new cases makes vertical control programs focused on single diseases less effective and efficient. Several districts are co-endemic for leprosy and BU. In these co-endemic districts and communities, the screening and management of these NTDs are very often carried out by the same health workers. To increase the effectiveness and efficiency of interventions and extend coverage of care in this context, a common integrated approach in the control and management of NTDs is recommended [3, 12, 13].

Theoretically, the notion of integration seems logical and simple, but its practical implementation remains a challenge because of traditional partnerships that have supported vertical control programs. For example, leprosy is supported by members of the International Federation of Anti-Leprosy Associations (ILEP) organization with their own modus operandi and highly vertical funding system. At the national and treatment facility levels, the technical staff is often different for each disease. From a technical point of view, each vertical program has its own strategic and normative plan, management tools, and notification and reporting system. Similarly, the interventions are sometimes specific to each disease depending on its mode of transmission and the technical guidelines for its management [different WHO technical guidelines]. Several publications on this concept of integration have emerged in recent years [12, 14–16]. Among these, Mitjà et al. proposed an integration approach based on 7 key points: initial assessment of the disease burden; training; development of an integrated control strategy for each district; social mobilization; active case detection; case management; and mapping and strengthening of health facilities.

Based on this approach, the two health programs, one in charge of the control of BU and the other the elimination of leprosy, experimented with the screening and the integrated management of cutaneous diseases in three co-endemic health districts of Côte d’Ivoire between April 2016 and March 2017.

We report here the results and lessons learned from the pilot test of this integrated approach to the detection and treatment of leprosy, BU and yaws associated with other skin diseases in three co-endemic health districts of Côte d’Ivoire, namely, Divo, Zouan-Hounien and Oumé.

## Method

### Study context

#### Buruli ulcer and leprosy programs in *Côte d’Ivoire*

Control activities for Buruli ulcer (BU) and leprosy are implemented by two different programs in Cote d’Ivoire:

- The National Program for the Elimination of Leprosy;
- The National Program for Buruli Ulcer Control.

There is no specific program for yaws eradication. Yaws management, as with other skin diseases, falls under the general health system.

The structures involved in the control of leprosy are organized nationally at three levels:

- *the central level:* with a coordinating office headed by an executive director of the National Programme for the Elimination of Leprosy and a leprosy referral center, namely, the Institut Raoul Follereau in Adzopé.
- *the intermediate level:* comprises 82 health districts with 82 chief medical officers. The screening, care and follow-up of patients are under the direct responsibility of leprosy-specialist nurses or of leprosy controllers based on the health departments or districts. Some leprosy-specialist nurses are responsible for several health districts, which explains the almost universal coverage of the country.
- *the peripheral level:* includes 1910 first-contact health facilities. In these institutions, 500 nurses and health workers trained to look out for signs of leprosy are responsible for detecting and referring all suspicious cases to the intermediate level for confirmation of diagnosis and then for caring for and following up with patients living in their jurisdiction.

For BU, the central level responsible for the administration, coordination and scientific support of the National Program for the Control of BU comprises the program’s Coordination Department, headed by a coordinating director, and six technical, administrative and financial services.

The intermediate level is composed of referral structures with adequate technical platforms, such as an operating theater for standardized case management. There are seven of these centers: two are public and five are faith-based centers.

It is thus apparent that the management of leprosy, BU, yaws and other common dermatological conditions is structured differently, which makes it difficult to develop an integrated strategy for their control.

### Study site and method

The study was conducted in the 3 health districts of Divo, Zouan-Hounien and Oumé in Côte d’Ivoire between April 2016 and March 2017. These districts are co-endemic for BU and leprosy. Localities in these 3 districts were targeted based on co-endemicity criteria.

The 3 health districts have different geographical and demographic characteristics. The District of Zouan-Hounien, with 210,453 inhabitants, is located in a mountainous region. The Districts of Divo and Oumé, which respectively have 404,821 and 296,670 inhabitants, are located in the middle of the Côte d’Ivoire forest belt.

From a health standpoint, the Districts of Zouan-Hounien, Divo and Oumé have 24, 42 and 25 peripheral health centers, respectively. The District of Divo has a regional hospital with a surgical unit dedicated to the management of complicated BU and leprosy cases.

This is a cross-sectional study using routine data that targeted populations in communities co-endemic for BU, leprosy and yaws in 3 districts of Côte d’Ivoire implemented between April 2016 and March 2017 in 6 stages: identification of potentially co-endemic communities; stakeholder training; social mobilization; mobile medical consultations; case detection and management; and a review meeting.

- Identification of co-endemic communities: During this phase, the co-endemic localities were identified through the analysis of available data.
  For leprosy and BU, this study used statistical data for the last five years available at the two programs. For yaws, the national statistical yearbook of 2015 [17] was used. It should be noted that these cases of yaws were reported essentially on a clinical basis without biological confirmation. As a result, 64 communities that are co-endemic for BU and leprosy were selected in the 3 districts: 10 localities in Divo, 34 in Zouan-Hounien and 20 in Oumé.
- Training of stakeholders: For the successful implementation of this approach, training on the three diseases was provided to 44 nurses and 50 community health workers. We trained nurses and community health workers in the integrated control and management of leprosy, BU and yaws. The training of the nurses covered basic epidemiology, clinical diagnosis, differential diagnosis, complications, social consequences, performing rapid diagnostic tests for yaws and treatment of these three diseases. The training for community health workers was mainly focused on clinical diagnosis to increase their capacity to identify possible cases.
- Social mobilization and sensitization: After the identification of the 64 communities, letters were sent to community leaders and to community radio for social mobilization. “Town criers” were also involved in announcing the event. These communities were visited one by the team. Sensitization kits including a generator, a sound system, a video projector and a projection screen were acquired for the implementation of sensitization activities. Some information, education and communication (IEC) materials for BU, leprosy and yaws were distributed. Movies on the diseases were presented to the population. Some comments were provided by the nurses.
- Mobile medical consultations: During this phase, five teams were formed according to the available experimented human resources for this activity. Each team was composed of qualified and specialized human resources in the fields of BU, leprosy and yaws (doctors, nurse specialists in dermatology, nurses, communication officers, etc.). These teams are experienced in the diagnosis and management of BU leprosy and yaws: they had been involved in the control of these diseases for several years. The consultations took place in schools in rooms well-lit by day light and ensured respect for the patient’s privacy. We included in the study all patients with skin signs and symptoms at the screening stage who voluntarily accepted screening by the team. These patients were then seen by nurses under the supervision of their trainers. They were carefully examined in a well-lit area that respected their privacy. The sociodemographic information and data on lesion characteristics were collected, as well as adequate samples to confirm BU and yaws cases. Only patients with skin lesions were included in this study. Patients who had general diseases without skin lesions were excluded and referred to the nearest health facility.
- Case detection and management: The leprosy and BU screening were performed according to the WHO clinical criteria. For BU cases, swabs or fine needle aspiration were collected accordingly by experienced nurses. BU cases were confirmed by polymerase chain reaction (PCR) for *IS2404* at the Pasteur Institute of Côte d’Ivoire. BU lesions were classified according to the WHO categories: Category I (a single lesion with a diameter of 5 cm); Category II (a single lesion with a diameter between 5 and 15 cm); and Category III (a single lesion with a diameter > 15 cm; multiple lesions; osteomyelitis; a lesion located in a critical area such as the eyes, breasts or genitals) [18].
  The yaws screening was performed on the basis of clinical suspicion and confirmed by two rapid tests. The first test was SD Bioline Syphilis 3.0; then, patients who tested positive were confirmed by DPP® Syphilis Screen & Confirm Assay. Cases of leprosy were diagnosed clinically according to the WHO clinical definition [19, 20] by specialist nurses with many years of experience in leprosy control. Cases were classified as paucibacillary (≤ 5 lesions) and multibacillary (> 5 lesions or with nerve involvement (pure neuritis, or any number of skin lesions and neuritis)) according to the WHO Global Leprosy Strategy 2016–2020 [19, 20].

All patients identified benefited from free treatment on-site within the community. After receiving the initial treatment onsite, patients with complicated cases were referred to the peripheral health center or to the specialized referral facility. New leprosy cases received multidrug therapy.

BU cases were referred to the health center for treatment. Former BU and leprosy patients attended counseling sessions for the prevention of impairment or disability. Some were given Vaseline (petroleum jelly) or shea butter (a locally available alternative to Vaseline) for the maintenance of their scars and prevention of skin dryness.

All cases of yaws were treated with azithromycin free of charge. Adequate treatment was given for the other skin conditions; this was most often antifungal medicines, soap, scabicides, Vaseline (petroleum jelly) or shea butter as indicated. The necessary inputs for bandages and dressings were also made available to patients.

- The review meeting: This meeting brought together all the stakeholders at the health district level, thus allowing them to review the process, give feedback to the health authorities, organize follow-up of the identified cases, and analyze the strengths and weaknesses as well as lessons learned.

### Statistical analysis of the data

All study data were recorded and processed with the software Microsoft Excel 2007. The frequencies of the different pathologies detected were calculated. The SWOT (strength, weakness, opportunity and threat) analysis was conducted by 4 doctors of the Buruli ulcer program and two nurse specialists in leprosy. This staff has experience with community-based disease control interventions. The SWOT matrix was used to analyze the strengths and weaknesses of the activity.

## Results

**Fig. 1:**
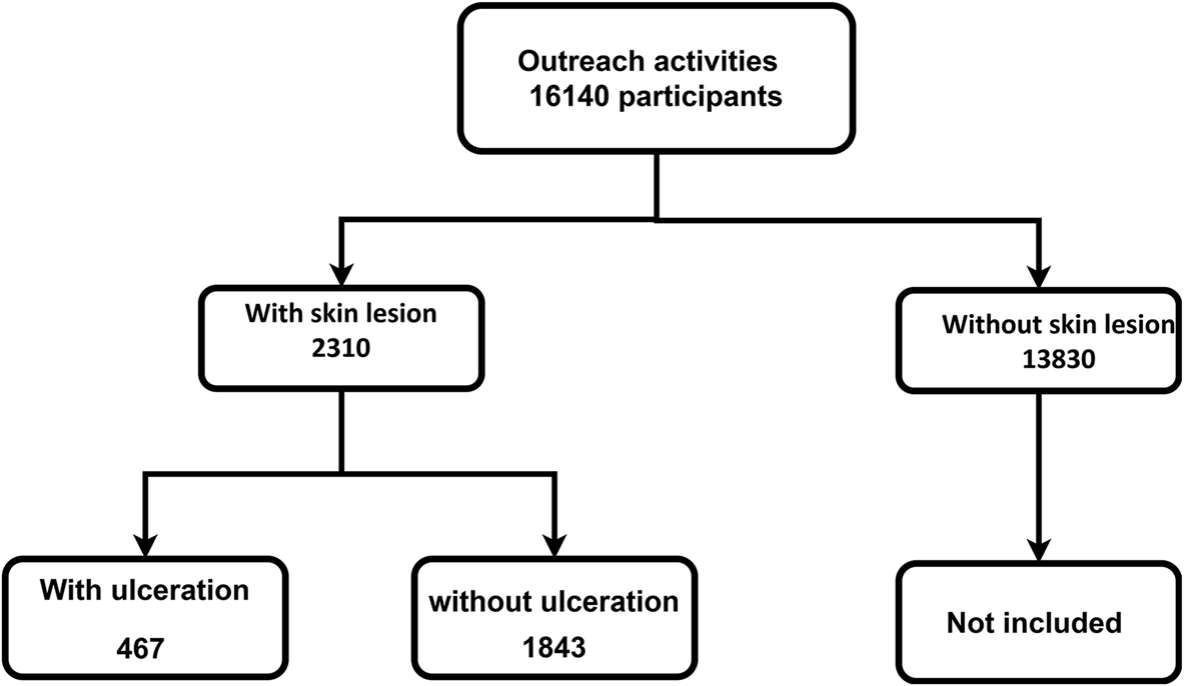
Flow chart of cases

This activity took place in 64 targeted locations across all 3 health districts co-endemic for BU and leprosy. The outreach activities were attended by 16,140 people (Fig. 1).

Within the sensitized population, 2310 (15%) had skin lesions (467 with ulceration and 1843 without ulceration): 1302 cases in the district of Divo, 566 cases in the district of Oumé and 442 cases in the district of Zouan-Hounien. The median age (IQR) of the participants with skin lesions was 13 years (9.5; 31); a majority of them (61.65%) were female.

Seven cases were diagnosed as Buruli ulcer; 5 out 7 cases were category II. There were 30 leprosy cases, and 17 cases (56.7%) were female. The median age (IQR) was 53 years (39.5; 69); 21 were paucibacillary and 09 were multibacillary. There were 15 cases of yaws. In total, 467 (20.22) patients had ulcerative lesions. Most of the ulcerative lesions were posttraumatic (11.90%), when nearly half of the patients had nonulcerative lesions (42.77%) and had fungal infection.

A summary of the main results is presented in Table 1.

**Table 1:** Detailed results of the integrated screening campaigns

Form of the dermatosis	Etiology	Divo (n)	Oumé (n)	Zouan-Hounien (n)	n (%)
**With ulceration**	Buruli ulcer	2	2	3	7^a^ (0.30)
	Yaws	15	0	0	15^b^ (0.65)
	Posttraumatic ulcerations	137	74	64	275 (11.90)
	Ulcerations of vascular origin	11	5	4	20 (0.87)
	Necrotizing fasciitis	35	27	24	86 (3.72)
	Post-erysipelas ulceration	5	3	4	12 (0.52)
	Eczema secondary infection	10	8	5	23 (1.00)
	Staphylococcal infection	12	9	8	29 (1.26)
	**Total**	**227**	**128**	**112**	**467 (20.22)**
**Without ulceration**	Leprosy	12	8	10	30^c^ (1.30)
	Fungal infection	601	220	167	988 (42.77)
	Eczema and prurigo	310	137	105	552 (23.90)
	Scabies	105	49	33	187 (8.10)
	Scratch injury	12	4	4	20 (0.87)
	Acne vulgaris	26	17	10	53 (2.29)
	Vitiligo	2	0	1	3 (0.13)
	Ichthyosis	3	1	0	4 (0.17)
	Lichen planus	3	1	0	4 (0.17)
	Neurofibromatosis	1	1	0	2 (0.09)
	**Total**	**1075**	**438**	**330**	**1843 (79.78)**
**Total**		**1302**	**566**	**442**	**2310** (**100.00**)

*n* number of patients

^a^ Catégorie II: 05 cases and Catégorie III: 02 cases

^b^ Eight cases diagnosed by rapid test (RDT) and confirmed by DPP; 07 cases diagnosed clinically

^c^ Paucibacillary: 21 cases and multibacillary: 09 cases

The SWOT analysis indicated that the integration of activities is possible and benefits the population and the commitment of partners. Human resources are available and can take care of some common skin diseases in their health zone, provided that they benefit from capacity building and the availability of necessary consumables for the care and follow-up of identified patients. However, the increased workload resulting from the implementation of this approach, requiring front-line health workers to examine and test all skin lesions, could be a major threat (Table 2).

**Table 2:** SWOT analysis

**STRENGTHS**	**WEAKNESSES**
• The motivation and enthusiasm of the actors favor their involvement in the implementation of the activities • The skills and experience gained by community actors in the control of BU and leprosy can be used to implement an integrated approach • The existence of a community volunteer network promotes social mobilization and case detection • The existence of databases on BU and leprosy can be used to determine co-endemic communities • The existence of health centers near the targeted localities helps to ensure the care and follow-up of the cases detected • The existence of referral health facilities with an adequate technical platform makes it possible to ensure the medical-surgical management of the referred complicated cases • Existence of a network of community health volunteers for the control leprosy and BU in every district in Côte d’Ivoire • The partners’ commitment to an integrated approach in the management of skin NTDs	• The lack of dermatologists makes the diagnosis and management of certain dermatoses difficult • Health personnel and community volunteers are not adequately equipped to diagnose and suspect dermatoses other than leprosy, BU and yaws • The lack of integrated case-reporting tools does not allow for standardized reporting • The increased workload for health personnel, which could jeopardize the sustainability of the integration
**OPPORTUNITIES**	**THREATS**
• The existence of the Regional Strategy on Neglected Tropical Diseases in the WHO African Region 2014–2020 [3] • The national interest in the control of NTDs through the creation of health programs in charge of the management of Buruli and leprosy • The possibility of intersectoral collaboration with the education sector for screening in schools	• Very broad theme: the range of skin diseases with cutaneous is indeterminate • The unavailability of dermatological cream at the level of first-contact health facilities, which could jeopardize the sustainability of routine dermatological consultations in health centers

## Discussion

In this study, we share our experience in implementing integrated management of skin NTDs in Côte d’Ivoire. Through this experiment, we think that the integrated screening and care of patients with skin NTDs is feasible in Côte d’Ivoire. Indeed, in recent years, the number of cases of leprosy and BU has considerably decreased in Côte d’Ivoire, as in most African countries, and the WHO has provided a training guide for front-line health workers [21]. These diseases are often co-endemic and show similar clinical signs, and financial and human resources to control them are limited. The implementation of this integrated approach in Côte d’Ivoire was organized using the tools and the human and material resources acquired as part of the efforts to control BU and leprosy. This implementation allowed us to detect and provide care for 7 cases of BU (0.3%) and 30 cases of leprosy (1.3%), of which 21 were paucibacillary and 9 were multibacillary. Fifteen cases of yaws were detected, and 8 were confirmed by serological testing; the other 7 were diagnosed on a clinical basis because we did not have rapid screening tests during the first campaign. It is possible that one or more of those cases were syphilis instead of yaws since previous studies showed that experienced clinicians did not have their diagnosis confirmed by laboratory tests [22]. In addition, there is a study showing syphilis in children acquired from nonsexual contact, and DPP is not useful to differentiate syphilis of yaws, which is also caused by *Treponema pallidum*, but in this case, *T. pallidum spp. Pertenue* [23]. Both diseases can be treated efficaciously by a single dose of azithromycin [24, 25].

The proportion of leprosy cases detected in our study is similar to the finding of earlier studies also conducted in Cote d’Ivoire, in Benin [26, 27] and in Malawi [28]. For these integrated activities, the most endemic districts were visited. Therefore, we cannot extrapolate our results to the population nationwide.

Apart from skin NTDs, many other skin diseases (97.75%) were detected and treated. This confirms one of the results of Msyamboza et al., who also noticed that the actors involved in the management of leprosy had acquired the ability to detect many other skin diseases [28]. In addition, to launch the series of campaigns, the nurses in the targeted health areas, who were already experienced in the management of BU and leprosy and were available, benefited from capacity building. This theoretically made them better equipped to diagnose and care for people with other skin conditions. The effect of this activity could be measured through their active and operational involvement during the mobile consultation sessions. These health professionals were able to accurately identify and adequately manage various skin diseases, as indicated in the results of our study. For example, only 7 cases were referred to a higher level for better care. Those were mainly chronic wounds of various etiologies as well as some cases of yaws or dermatomycosis. Eight cases of yaws that were detected by the nurses and confirmed positive by the rapid test during the last campaign provide evidence for the quality of the diagnosis.

What is the degree of ownership of this integrated approach among the different actors in the health system and in the community in Côte d’Ivoire? According to the WHO, institutional ownership is an important component of the integrated management of NTDs. It is one of the four priority strategies that the WHO impresses upon all countries as part of the development of NTD master plans [29]. Furthermore, a resolution of the WHO Regional Office for Africa also recommends that African countries promote leadership to establish and strengthen national integrated NTD programs and to promote multisectoral collaboration [30]. The mobilization of health system stakeholders and community actors resulted in good planning and successful implementation of the integration. As a result, each active phase of consultations was preceded by social mobilization. The approval and accession of the health authorities for our study were reflected in the active participation of the programs responsible for coordinating the control of BU and leprosy. These two structures attached to the Directorate General for Health in Côte d’Ivoire demonstrated their approval not only by disseminating technical notes and signing the terms of reference for each activity but also by actively participating in the implementation of the campaigns in collaboration with the local health authorities. The involvement and participation of the community were important assets in the management of BU [31, 32]. The implementation relied on this model. During the activities, mobilized community leaders helped to reach the participants. Just as in the management of BU and leprosy, they are a human resource that can be mobilized to sustain the integrated management of NTDs.

What are the major difficulties and constraints of this strategy? The SWOT analysis performed prior to the implementation of the integrated approach enabled us to measure the possible threats and weaknesses in the implementation of such activities. Although the planning of activities took into account the WHO guidelines, difficulties and constraints were identified. They are linked to the mobilization of logistical assets and to the geographical accessibility of targeted co-endemic localities. Indeed, mobile consultations require heavy logistical loads to be transported to localities on access roads that are difficult to pass. However, the people most affected by NTDs live in remote areas that sometimes lack easy access to the health system [16]. Because of the remoteness of locations and the logistical challenges of arriving there, preventive chemotherapy for leprosy and yaws could be administered when the campaign is conducted. The same opportunity could be used for education and/or other interventions aimed at reducing stigma towards persons with leprosy, BU and yaws.

In addition to health professionals, community health workers were trained in the recognition of suspicious cases and in social mobilization. It is certainly difficult to assess the impact of these sessions on the behavioral changes in the population; however, we know that the presence of children (50%) and women (45%) is linked to sensitization sessions (these two categories are the most vulnerable to and the most affected by NTDs). According to the WHO, “many neglected tropical diseases affect women and children disproportionately. Those living in remote areas are the most vulnerable to infections and to their biological and socio-cultural consequences.”

It should be noted, however, that implementation requires the mobilization of human resources as well as the analysis and definition of the array of activities to be carried out, as recommended by Mitjà et al. [15]. In fact, the campaigns we conducted in Côte d’Ivoire took into account that recommendation by including sensitization, screening and patient care in our integrated approach. The logistical assets and the necessary drugs and inputs were mobilized. Moreover, the majority of screened cases were taken care of within the community. Subsequently, the follow-up of these cases was carried out by nurses in the peripheral health centers.

The model of the integrated approach in the management of NTDs that we tested had ethical limits. Several people with different health problems gathered in the same place to attend mobile clinics. This had the theoretical advantage of breaking down barriers of stigma; however, in practice, it can also create a recruitment bias since people with very advanced lesions or elderly people may not present themselves in public. However, during our study, there were instances where teams did home-based consultations on the indication of community health workers. Another limitation of this study is the selection of patients with skin lesions. Patients with primary neural leprosy (PNL) or very tiny or scanty lesions may be missed. To our knowledge, there are no data on the prevalence of primary neural leprosy in Côte d’Ivoire. A study in India reported it to be 4 to 18% [33]. Some authors think that it is less common in Africa and have mentioned a prevalence less than 1% [34]. Therefore, we think that the number missed is likely to be very small. Future studies might focus on what kind of strategy can be planned to avoid missing those cases. In addition, we did not detect leprosy cases among children during this activity. It will be useful to continue the survey to confirm the absence of leprosy cases in children in these localities.

Since it is the first time we are conducting these activities in Côte d’Ivoire, the effectiveness of the calls for the community to participate in this campaign was not measured at this time. The next study will focus on this aspect.

The other constraint that must be overcome to ensure the sustainability of this integrated approach is the free accessibility or at least the affordability of drugs for other diseases similar to BU, yaws and leprosy. Most dermatological drugs are on the list of essential drugs in Côte d’Ivoire but are not available in first-contact facilities. Patients will come only when they have easy access to medications. Financial accessibility makes it easy to access health care and allows better management of the disease through the use of health facilities from the earliest symptoms of illness and the availability of medications [35]. Some cases of diagnostic difficulties by nurses were noted; such cases were few. For example, the two cases of neurofibromatosis were diagnosed by supervising physicians and were referred for treatment. To address such cases, it is necessary to provide the support of a dermatologist or a nurse specializing in dermatology and leprosy during these interventions. Teledermatology consultations with dermatologists are also an alternative, and WhatsApp is a possible solution.

## Conclusions

Our study, based on the implementation of an integrated approach in the management of NTDs, took place in 3 districts in Côte d’Ivoire. The results of this study show that the integration of activities is possible given the national interest in the control of NTDs and the commitment of partners. Human resources are available and able to take care of some of the common skin diseases in their health area. It is essential that these human resources benefit from capacity building and have the necessary equipment for the care and follow-up of identified patients, including those with other dermatoses.

## Data Availability

The datasets used and/or analyzed during the current study are available from the corresponding author upon reasonable request.

## Abbreviations

BU: Buruli Ulcer
CM-NTDs: Case Management-Neglected Tropical Diseases
DPP: Dual Path Platform
ILEP: International Federation of Anti-Leprosy Associations
NTDs: Neglected Tropical Diseases
PC-NTDs: Preventive Chemotherapy-Neglected Tropical Diseases
PCR: Polymerase Chain Reaction
PNL: Primary Neural Leprosy
SWOT: Strengths Weaknesses Opportunities Threats
WHO: World Health Organization
